# Influenza virus intracellular replication dynamics, release kinetics, and particle morphology during propagation in MDCK cells

**DOI:** 10.1007/s00253-016-7542-4

**Published:** 2016-04-29

**Authors:** Timo Frensing, Sascha Y. Kupke, Mandy Bachmann, Susanne Fritzsche, Lili E. Gallo-Ramirez, Udo Reichl

**Affiliations:** Bioprocess Engineering, Max Planck Institute for Dynamics of Complex Technical Systems, Sandtorstrasse 1, 39106 Magdeburg, Germany; Chair of Bioprocess Engineering, Otto-von-Guericke University Magdeburg, Universitätsplatz 2, 39106 Magdeburg, Germany

**Keywords:** Influenza virus, Vaccine production, Virus release, Virus particle morphology

## Abstract

Influenza viruses are respiratory pathogens and can cause severe disease. The best protection against influenza is provided by annual vaccination. These vaccines are produced in embryonated chicken eggs or using continuous animal cell lines. The latter processes are more flexible and scalable to meet the growing global demand. However, virus production in cell cultures is more expensive. Hence, further research is needed to make these processes more cost-effective and robust. We studied influenza virus replication dynamics to identify factors that limit the virus yield in adherent Madin-Darby canine kidney (MDCK) cells. The cell cycle stage of MDCK cells had no impact during early infection. Yet, our results showed that the influenza virus RNA synthesis levels out already 4 h post infection at a time when viral genome segments are exported from the nucleus. Nevertheless, virus release occurred at a constant rate in the following 16 h. Thereafter, the production of infectious viruses dramatically decreased, but cells continued to produce particles contributing to the hemagglutination (HA) titer. The majority of these particles from the late phase of infection were deformed or broken virus particles as well as large membranous structures decorated with viral surface proteins. These changes in particle characteristics and morphology need to be considered for the optimization of influenza virus production and vaccine purification steps. Moreover, our data suggest that in order to achieve higher cell-specific yields, a prolonged phase of viral RNA synthesis and/or a more efficient release of influenza virus particles is required.

**Electronic supplementary material:**

The online version of this article (doi:10.1007/s00253-016-7542-4) contains supplementary material, which is available to authorized users.

## Introduction

Influenza is a contagious respiratory disease caused by influenza virus infections. Annually, influenza A and B viruses account for three to five million cases of severe illness and between 250,000 and 500,000 people decease due to these infections worldwide (estimates of the World Health Organization (WHO), Fact sheet Influenza No. 211, March 2014). Especially very young children (< 2 years), older people (> 65 years), and patients with underlying medical conditions such as chronic and metabolic diseases or a weakened immune system are at high risk to develop flu-related complications. Despite numerous attempts to develop drugs for the treatment of severe influenza infections, only two classes of antiviral drugs have been licensed so far. However, due to their frequent use, resistant influenza virus variants have already emerged. Thus, the best protection against influenza is annual vaccination. Nowadays, cell culture-based influenza vaccine production has become an important alternative to the conventional manufacturing process in embryonated chicken eggs. Compared to egg-based processes, cell culture technology has significant advantages such as higher flexibility and scalability since it is independent from the timely supply and laborious handling of embryonated eggs. In addition, these processes are not vulnerable to the threat of avian influenza viruses that can kill laying flocks. Moreover, sterility can easier be maintained in cell culture (reviewed by Audsley and Tannock (2008)). Recently, the first recombinant influenza vaccine produced in insect cells received approval (Buckland et al. 2014). Other vaccine manufacturers propagate influenza viruses in MDCK cells or African green monkey kidney (Vero) cells. However, a thorough understanding of the viral replication cycle, virus-host cell interactions, and virus spreading in cell populations is crucial to further optimize vaccine production in these cells.

Influenza viruses belong to the family of *Orthomyxoviridae*. They are enveloped viruses with a segmented single-stranded RNA genome of negative polarity. The genome of influenza A viruses consists of eight segments encoding for 10 major proteins and additional seven accessory polypeptides (reviewed by Vasin et al. (2014)). Each genomic viral RNA (vRNA) segment is present as a viral ribonucleoprotein (vRNP) complex containing the tripartite RNA-dependent RNA polymerase (RdRP) complex and the vRNA encapsidated by multiple monomers of the viral nucleoprotein (NP). The RdRP accomplishes both transcription and replication of the viral genome, which takes place in the nucleus of infected cells. While transcription is directly initiated after the nuclear import of vRNPs, replication can only proceed in the presence of newly synthesized viral proteins. It has been reported that newly produced RdRPs and NPs stabilize the replication intermediates, i.e., complementary RNAs (cRNAs), forming cRNPs which serve as templates for the synthesis of progeny vRNAs (reviewed by Elton et al. (2006)). At later stages of the infection cycle, the matrix protein 1 (M1) and the nuclear export protein (NEP; also known as non-structural protein 2 (NS2)) bind to vRNPs which leads to their nuclear export and to the termination of viral RNA synthesis (reviewed by Cros and Palese (2003)). Subsequently, viral genomes and proteins are transported to the plasma membrane where virus assembly and budding take place (reviewed by Bouvier and Palese (2008)). Activation of signal transduction pathways and accumulation of viral components in the course of an infection lead to the induction of programmed cell death (apoptosis). On the one hand, virus propagation can be impaired when apoptosis is induced early during infection before virus replication has reached its full magnitude. On the other hand, apoptosis at later time points seems to support the release of virions and therefore becomes pro-viral (reviewed by Herold et al. (2012)). Many details of the influenza virus life cycle have already been unraveled, but still little is known about the relative importance of all steps involved and how their interplay determines the virus titer in cell cultures.

To identify possible bottlenecks for the production of influenza viruses on the molecular level, we thoroughly studied the dynamics of influenza virus replication in adherent MDCK cells. To this end, we analyzed virus release kinetics in a single-cycle infection by exchanging the medium in regular intervals. Using electron microscopy, we compared the morphology of virus particles from an early phase of the infection with particles from the later stage when almost exclusively noninfectious particles were produced. In addition, we examined the intracellular dynamics of viral RNA synthesis and the localization of viral components in the course of an infection cycle. The latter was done by imaging cytometry that combines the throughput and sensitivity of conventional flow cytometry with the spatial resolution of fluorescence microscopy. Finally, we performed an infection at low multiplicity of infection (MOI) to investigate if cells in a particular cell cycle stage become preferentially infected and support enhanced influenza virus replication.

## Materials and methods

### Cells and viruses

Adherent MDCK cells (ECACC, No. 84121903) were cultivated in Glasgow’s minimum essential medium (GMEM), supplemented with 10 % (*v*/*v*) fetal calf serum (FCS) and 1 % (*v*/*v*) peptone at 37 °C in a 5 % CO_2_ atmosphere. For infection experiments, we used serum-free infection medium comprised of GMEM, 1 % (*v*/*v*) peptone and trypsin (Sigma-Aldrich, # T7409) was applied at a concentration of 5 BAEE U/mL (4.5 μg/mL). Influenza virus strain A/Puerto Rico/8/34 (PR8) was obtained from the National Institute for Biological Standards and Control (No. 06/114). The seed virus titer was determined by standard plaque assay (1.76 × 10^8^ plaque forming units (PFU) per mL).

### Virus infections

For single-cycle experiments confluent cells in T75 flasks were washed twice with phosphate-buffered saline (PBS) and afterwards infected at an MOI of 10 PFU per cell in 3 mL of infection media for 1 h. During the incubation at 37 °C in a 5 % CO_2_ atmosphere, flasks were rocked every 20 min to keep the monolayer moist and to distribute viruses evenly. Thereafter, the inoculum was removed, cells were washed twice with PBS, and 13 mL of infection media were added to the flasks. At every sampling time point, one flask was used to analyze the intracellular infection dynamics by quantitative reverse transcription PCR (RT-qPCR) and imaging flow cytometry.

To study virus release kinetics, only 5 mL of virus infection media were added to one flask. The supernatant of this flask was harvested every 4 h. To this end, the flask was rocked to swirl up detached cells and the supernatant was harvested. Five milliliters of infection media was used to wash the cell monolayer and was then combined with the supernatant. Fresh infection media (5 mL) was added to the flask that was returned to the incubator. We then determined the cell number in the supernatant (i.e., detached cells) using a Vi-CELL™ (Beckman Coulter), the remaining sample was centrifuged at 300×*g* for 10 min at 4 °C. Aliquots of supernatants were stored at −80 °C until virus titration. Four flasks containing 13 mL of infection media served as controls to determine the virus titer without medium exchange and to obtain the cell count of adherent cells.

In addition, low MOI infections were performed to investigate if cells in a certain cell cycle stage become preferentially infected. One day before infection, 2.5 × 10^6^ MDCK cells were seeded in T25 flasks and incubated at 37 °C and 5 % CO_2_ for 14 h. Thereafter, the cells were mock-infected or infected with influenza virus PR8 at an MOI of 0.1 in 1 mL infection medium. The inoculum was removed after 45 min, cells were washed once with PBS, and cells were incubated at 37 °C and 5 % CO_2_ in 3 mL GMEM supplemented with 10 % (*v*/*v*) FCS (without trypsin to reduce secondary infections). At each sampling time point, one T25 flask was harvested.

### Virus quantification

Virus titers were determined by the hemagglutination assay (Kalbfuss et al. 2008) and the 50 % tissue culture infective dose (TCID_50_) assay (Genzel and Udo 2007). Titers of the HA assay were expressed as log_10_ HA units per test volume (log_10_ HAU/100 μL). Total virus particle concentration (*c*_virus_ [virions/mL]) was determined, assuming that agglutination occurs up to a dilution in which the amount of virus particles equals the amount of erythrocytes (Werner and Schlesinger 1954). Thus, the calculation was based on HA titer and cell concentration of the erythrocyte suspension (2 × 10^7^ cells/mL).

$$ {c}_{\mathrm{virus}}=2\times {10}^7\times {10}^{\left({ \log}_{10}HAU/100\upmu \mathrm{L}\right)} $$

Cell-specific, cumulative virus release was assessed by referring to the maximum cell count obtained in each experiment.

### Sampling for RT-qPCR and imaging flow cytometry

Infected cells in T75 flasks were rocked to swirl up detached cells and the supernatant was harvested. Detached cells were separated from the supernatant by centrifugation. Remaining adherent cells in T75 flasks were trypsinized and afterwards combined with the detached cells from the previous step. For this cell suspension, we then determined cell count and viability (by trypan blue staining) using a Vi-CELL™ (Beckman Coulter). An aliquot of 1 × 10^6^ cells was centrifuged and cell pellets were lysed with 350 μL of lysis buffer RA1 (Macherey Nagel) containing 1 % (*v*/*v*) β-mercaptoethanol. Lysates were stored at −80 °C until RNA purification according to the manufacturer’s instructions (“NucleoSpin RNA” from Macherey Nagel). The remaining cell suspension was fixed with paraformaldehyde at a final concentration of 1 % (*w*/*v*) and aliquots of 1 × 10^6^ cells were stored in 70 % ethanol at −20 °C until staining for imaging flow cytometry. For infection experiments at low MOI, 2 × 10^6^ cells were collected and fixed for the analysis by imaging cytometry.

### In vitro synthesis of RNA reference standards

For absolute quantification of intracellular v/c/messenger RNA (mRNA) concentration of segment 5, corresponding RNA reference standards were synthesized by in vitro transcription. For this, plasmids carrying the full sequence of v/c/mRNA were used as template for conventional PCR. The T7 promotor sequence was introduced by primers (Online Resource, Tab. S1). PCR conditions were as followed: denaturation at 98 °C for 3 min, 35 cycles at 98 °C for 25 s, 53 °C for 45 s, and 72 °C for 90 s and final elongation at 72 °C for 10 min. Thereafter, PCR products were purified (InnuPrep PCR pure Kit, Analytik Jena) and used for in vitro transcription (TranscriptAid T7 High Yield Transcription Kit, Thermo Fisher Scientific) according to the manufacturer’s instructions. Finally, RNA standards were purified (NucleoSpin RNA Clean-up Kit, Macherey & Nagel) and stored at −80 °C.

### Real-time RT-qPCR

In order to quantify intracellular levels of v/c/mRNA, we used a real-time RT-qPCR approach with polarity- and gene-specific tagged primers in the RT reaction. Primer sequences are listed in supplementary table S2 (Online Resource). In brief, 1 μL of RNA was mixed with 1 μL primer (1 μM for v/cRNA; 10 μM for mRNA), 1 μL dNTPs (10 mM each), and 11.5 μL RNase/DNase-free water. Thereafter, the mixture was incubated at 65 °C for 5 min and subsequently cooled to 42 °C for mRNA and 55 °C for c/vRNA. The reaction mixture (4 μL 5X Reaction Buffer, 0.5 μL Maxima H-Minus Reverse Transcriptase (200 U/μL, Thermo Scientific), 0.5 μL RiboLock™ RNase Inhibitor (40 U/μL, Thermo Scientific), 0.5 μL RNase/DNase-free water) was pre-warmed at 42 °C for mRNA and 55 °C for c/vRNA for 1 min, added to the RNA reaction mix and incubated at 60 °C for 30 min before a final RT inactivation step at 85 °C for 5 min was carried out. In addition, RT reactions were performed with 10-fold serial dilutions of the corresponding reference standard (5 ng to 5 × 10^−7^ ng). Thereby, 350 ng of total MDCK RNA was added to mimic intracellular conditions within the standard samples. Finally, complementary DNA (cDNA) was filled up to 100 μL with RNase/DNase-free water and stored at −20 °C until use. Viral RNA concentrations were determined with the “Rotor-Gene SYBR Green PCR Kit” (QIAGEN) by real-time RT-qPCR. To this end, 4 μL of cDNA, 1 μL primer set (Online Resource, Tab. S3) and 5 μL of reaction mixture were combined and analyzed with a Rotor-Gene Q real-time PCR cycler (QIAGEN). The temperature profile included denaturation at 95 °C for 5 min, amplification in two steps at 95 °C for 10 s, and 62 °C for 20 s and a melting curve from 65 to 90 °C. To calculate viral RNA concentration, C_t_ values obtained for the RNA reference standards were plotted against log_10_ numbers of viral molecules resulting in a linear calibration curve. The number of viral RNA molecules (*n*_molecules_) was determined based on quantity of the template (*m*_template_ [ng]), fragment length (*N*_(bases)_ [bp]), average mass of one base (*k* = 340 [Da/bp] and the Avogadro constant (*N*_A_[mol^−1^]).

$$ {n}_{\left(\mathrm{molecules}\right)}=\frac{m_{\mathrm{template}}}{k\times {N}_{\left(\mathrm{bases}\right)}\times {N}_{\mathrm{A}}^{-1}\times {10}^9} $$

To calculate the number of molecules per cell (SQ_sample_ [molecules/cell]), the slope (m) and the *y*-intercept (b) of the calibration curve, the coefficient for dilution of RT reaction (F_RT_) and the volume of RNA eluate (*V*_eluate_ [μL]) were considered and finally referred to the cell number (*n*_cells_).

$$ {SQ}_{\mathrm{sample}}=\frac{10^{\left(\frac{Ct-b}{m}\right)}\times {F}_{RT}\times {V}_{\mathrm{eluate}}}{n_{\mathrm{cells}}} $$

### Imaging flow cytometry

Stored samples were centrifuged for 10 min at 300×*g* and 4 °C and the supernatant was discarded. Then, cells were washed in 4 mL fluorescence-activated cell sorting (FACS) buffer (PBS, 2 % (*w*/*v*) glycine, 0.1 % (*w*/*v*) bovine serum albumin (BSA)) and centrifuged as before. Subsequently, cells were blocked in 150 μL FACS buffer containing 1.1 % (*w*/*v*) BSA for 1 h on ice. After centrifugation for 10 min at 300×*g* and 4 °C, cell pellets were resuspended in 100 μL antibody solution. All antibody incubations were performed at 37 °C for 1 h in the dark. The monoclonal mouse anti-NP antibody mAb61A5 (a kind gift from Fumitaka Momose) was used at a dilution of 1:500. This antibody preferentially binds to NP in the conformation inherent to the vRNP complex (Momose et al. 2007). Following incubation, the cells were washed three times with FACS buffer. Secondary antibody staining was performed using Alexa Fluor 647-conjugated polyclonal goat anti-mouse antibody (LifeTechnologies, #A21235) at a dilution of 1:500. Subsequently, cells were washed three times with wash buffer and 4′,6-diamidino-2-phenylindole (DAPI) was used for nuclear staining.

The immunostaining of M1 was performed using a FITC-conjugated monoclonal mouse anti-M1 antibody (AbD serotec, #MCA401FX) at a dilution of 1:100. After incubation and three washing steps, cells were resuspended in 40 μL of wash buffer. RNA degradation was conducted by adding 5 μL PureLink™ RNase A (20 mg/mL, life technologies). For nuclear staining, 0.5 μL of 7-AAD (Millipore) were added followed by an incubation for 30 min at room temperature in the dark.

Using the ImageStream X Mark II (Amnis, EMD Millipore) 10,000 single cells per sample (debris and cell doublets were excluded) were analyzed using ×40 or ×60 objective lenses. For infection experiments at low MOI, up to 300,000 single cells were measured. The 375 and 642 nm lasers were utilized for the excitation of the DAPI- and vRNP-stained samples. Channels 1 (CH1) and 5 (CH5) were acquired along with the bright field (BF) imagery on channel 6 (CH6). For the M1- and 7-AAD-stained samples, the 488 and 642 nm lasers were utilized for excitation and signal acquisition was conducted in channels 2 (CH2) and 5 (CH5) along with the BF imagery on channel 1 (CH1). Before acquisition, the laser power was adjusted to yield a “raw max pixel” feature value between 200 and 1500 of the single-stained positive controls. One thousand cells of these samples were acquired for compensation with the respective compensation settings.

### Image analysis

IDEAS software (version 6.1) was used for image analysis. Compensation matrices were generated using the corresponding compensation files. Only single cells in-focus were selected for analysis. Segmentation masks for M1- and vRNP-positive cells were generated based on mock-infected samples.

Nuclear localization of vRNPs as well as M1 was assessed by calculating fractions of fluorescence intensity (FI) of vRNP or M1 signal co-localized with the DAPI or 7-AAD signal, respectively. For vRNP analysis, the mask “nucleus” was generated using the function “morphology” (on CH1 imagery) and the mask “whole cell” was generated using the function “object” (on CH6). Features termed “intensity CH5 nucleus” and “intensity CH5 whole cell” were created by utilizing feature “intensity” (of CH5) within mask nucleus and whole cell, respectively. The combined feature “FI in nucleus” was generated with following definition: intensity CH5 nucleus/intensity CH5 whole cell. CH1- and Ch5-double positive cells were plotted on histograms using this feature. Mean values, multiplied by 100, yielded the fraction of FI in the nucleus (%). The M1 analysis was performed using the same procedure, but taking the corresponding channels into account.

Apoptotic cells were quantified based on chromatin condensation and nuclear fragmentation (leading to an increase in the intensity and a decrease in the area of the nuclear signal) as well as cell shrinkage (leading to higher BF contrast) as described before (Maguire et al. 2011). For this, the mask “threshold 50 % CH1” was generated using the function “threshold” on CH1 imagery (parameter “intensity percentage” at 50 %). A feature called “area threshold 50 % CH1” was generated by using feature “area” on mask area threshold 50 % CH1. Distinct populations of apoptotic cells were differentiated from non-apoptotic cells by plotting focused, single cells on feature “contrast CH6” against area threshold 50 % CH1.

The gates for cell cycle phases G_0_/G_1_, S, and G_2_/M were defined in a dot plot of DAPI intensity vs. DAPI signal area (Online Resource, Fig. S1). Infected cells were identified using a Max Pixel histogram of mock-infected samples for each time point.

### nsTEM analysis

An imaging service using negative stain transmission electron microscopy (nsTEM) was performed by Vironova (Stockholm, Sweden). Briefly, 3 μL of the sample (inactivated by β-propiolactone) were applied on glow discharged carbon-coated 400 mesh copper grids using the direct drop procedure. Samples were incubated for approximately 30 s, blotted off using a filter paper, washed with MilliQ water and finally negatively stained using 2 % uranyl acetate. After blotting off the stain, the grid was allowed to air dry. Grids were imaged using a Tecnai G2 Spirit BioTWIN (FEI) electron microscope operated at an accelerating voltage of 100 kV. Images were acquired using a 2 k × 2 k Veleta CCD camera (Olympus Soft Imaging System, OSiS).

For each sample, five remotely located grid positions, with good sample embedding, were selected. At each position, by traversing the grid at 68 k magnification, a total of 20 images were acquired. Image data was collected, as soon as one or more particles were detected in the field of view. Particles were subsequently classified as intact, deformed, or broken, and large membranous by manual image analysis. Thereby, spherical and close to spherical particles approximately 80–140 nm in diameter with well-resolved surface spike proteins were classified as intact particles. Particles that displayed major perturbations and even rupture of the membrane were classified as deformed or broken particles. Moreover, particles that had the characteristic appearance of lipid membranes (folded structures, commonly with bright edges and a denser interior) and were larger than the typical influenza virion in size (200–700 nm) were classified as large membranous particles.

## Results

### Production dynamics of influenza viruses in MDCK cells

**Fig. 1:**
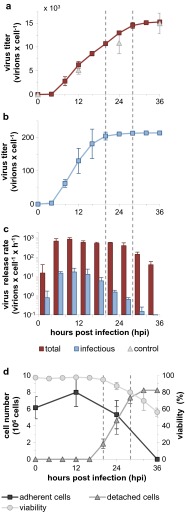
Virus production kinetics. MDCK cells were infected with the influenza virus PR8 at an MOI of 10 PFU per cell. The supernatant was harvested every 4 h and replaced with fresh media. Infections without medium exchange (incubated for 12, 24, and 36 h) served as controls. The number of total virus particles was calculated based on the HA titer and infectious virions were quantified by the TCID_50_ assay. Depicted are the cumulative titers of total (**a**) and infectious virus particles per cell (**b**) as well as the virus release rates per hour (**c**). The *first and the second dashed lines* indicate time points when ≥95 % of the final titer of infectious and total particles were released, respectively. Control titers (*upper panel*) indicate the number of total virions released from infected cells without medium exchange. **d** Cell counts and cell viability. The cumulative numbers of detached cells are depicted. Numbers of adherent cells and viabilities of all cells (based on trypan blue staining) are derived from infected cells without medium exchange. *Error bars* indicate standard deviation of three independent experiments

To study thoroughly the dynamics of influenza virus replication in cell lines used for vaccine manufacturing, we infected MDCK cells with the influenza strain A/Puerto Rico/8/34. In contrast to vaccine production processes which typically use a low MOI, we performed the infection at an MOI of 10 PFU per cell. This high MOI ensures that the complete cell population becomes rapidly infected and consequently enables the detailed analysis of a synchronized single-cycle infection. We investigated the dynamics of virus production by harvesting the complete supernatant of the cells and subsequent addition of fresh medium every 4 h. All samples were subjected to the TCID_50_ assay and the HA assay to determine the concentration of infectious and total virus particles, respectively. Cumulative growth curves were generated by adding up the titers of every successive sampling interval in the course of the infection experiment (Fig. 1a, b). To check whether the replacement of the medium every 4 h had an impact on the infection process, cells were infected under the same conditions but incubated without medium exchange for 12, 24 and 36 h. No significant differences in the total virus particle counts were observed between cells with and without medium exchange demonstrating that the experimental procedure did not affect the influenza virus propagation. Under both conditions, the same final HA titer was reached which corresponded to approximately 15,000 particles per cell (Fig 1a). In contrast, infectious virus particles reached lower final yields of about 200 virions per cell (Fig. 1b). When we calculated the cell-specific virus particle production per hour, it became obvious that the rate of virus release for both infectious and total virus particles reached its maximum already in the sampling interval between 4 and 8 h post infection (hpi) (Fig. 1c). In the following 12 h, the virus release occurred at a rather constant rate and the ratio of infectious to total virus particles was more or less stable. However, the production of infectious particles started to decline already between 16 and 20 hpi and 95 % of the final infectious virus yield was reached at 20 hpi (first dashed line). In contrast, a significant drop in the release of total particles detected by the HA assay occurred after 28 hpi when 95 % of the final total virus particle count was reached (second dashed line). In the early phase (before 20 hpi), 2 to 4 % of the released influenza virus particles were infectious. At later stages of the infection, cells almost exclusively produced noninfectious particles (below 0.3 % infectious particles). In addition, we determined cell counts and the cell viability in the time course of the single-cycle infection. Simultaneous to the decrease in the production of infectious particles, the adherent MDCK cells started to detach from the bottom of the cell culture flask and the viability measured by trypan blue staining started to decrease (Fig. 1d). Most of the cells were found in the supernatant at 28 hpi when the production of total virus particles also declined. Taken together, our thorough analysis of the single-cycle infection experiment revealed that progeny virus particles are released with a rather constant rate for a relatively long period of time. However, the production of infectious particles decreased much earlier than the production of particles contributing to the HA titer.

### Morphology of influenza virus particles from early and late stages of the infection

**Fig. 2:**
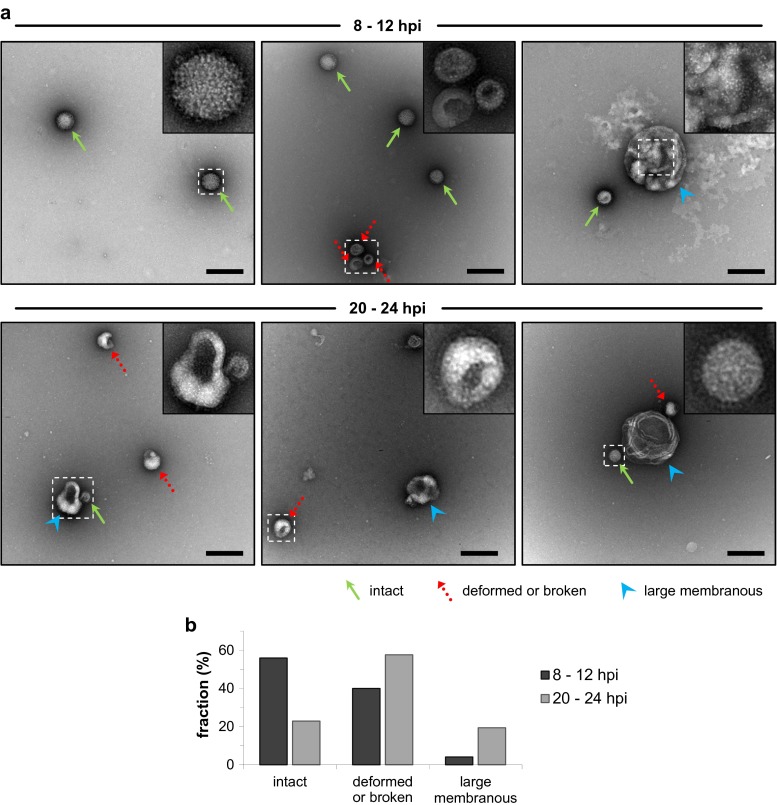
Virus particle morphology at early and late stages of virus production. Cells were infected with influenza virus PR8 at an MOI of 10 PFU per cell and supernatants were harvested and replenished with fresh medium every 4 h. Selected samples were subjected to negative stain transmission electron microscopy. **a** Virus particle morphology. Representative images of released particles at early (8–12 hpi) and late (20–24 hpi) stages of virus production are shown. Particles were classified as indicated by *arrows*: (I) intact and spherical with well-resolved surface spike proteins; (II) deformed or broken with distinct spike protein-decorated surface; (III) large membranous, of which many appear spike-decorated. *Insets* show higher magnifications of selected particles. *Scale bar* indicates 250 nm. **b** Quantitative results of particle classification. Particles of a total of 100 images per sample (yielding 345 and 314 particles for early and late stages, respectively) were subjected to manual classification analysis. Imaging data of one representative experiment is depicted (two independent experiments were analyzed)

Next, we investigated differences between virus particles produced at early time points compared to late time points when almost exclusively noninfectious particles were produced. To this end, particle morphology was examined by nsTEM. In the early phase of the infection (between 8 and 12 hpi) most particles were spherical (Fig. 2a, upper panel). In addition to these apparently intact virus particles, deformed or broken particles and large membranous structures were present. The latter often appeared decorated with viral surface proteins. The average diameter of 435 particles from the early phase of an infection was 117 ± 25 nm (data not shown). The proportion of particles with the different afore mentioned morphologies was quantified by analyzing 100 nsTEM images of a sample of an early time frame (8–12 hpi) and 100 images of a sample representing the time frame 20 to 24 hpi. The particle classification clearly showed that more than 50 % of the virions appeared as intact spherical particles at the early sampling time point (Fig. 2b). In contrast, only about 25 % of the particles were intact spherical at the later stage between 20 and 24 hpi. At this stage, the majority of particles was deformed or broken and the amount of large membranous structures also increased. Thus, the particle characteristics change during the course of one infection cycle and the morphological analysis supports the previous observation that less infectious particles are produced at later stages of the infection (Fig. 1).

### Dynamics of the influenza virus RNA synthesis

**Fig. 3:**
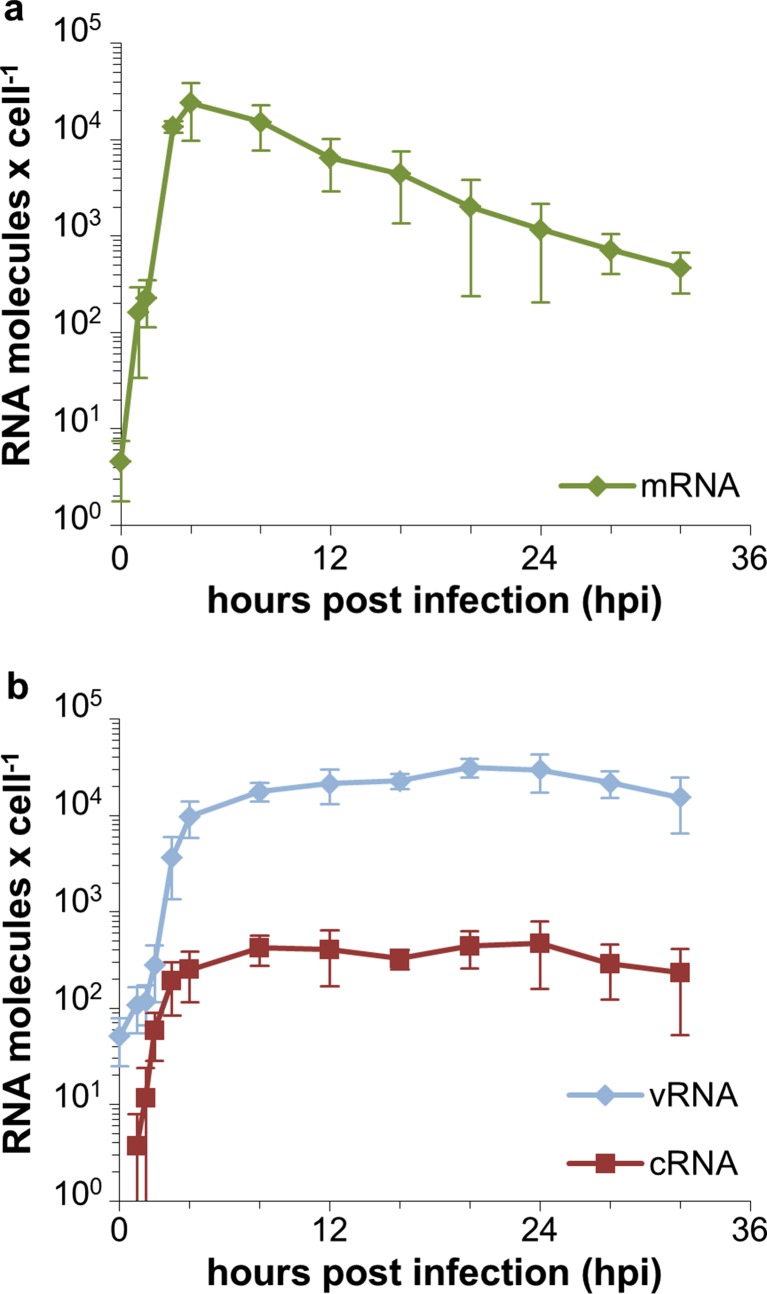
Dynamics of the intracellular viral RNA synthesis. MDCK cells were infected with the influenza virus PR8 at an MOI of 10 PFU per cell and harvested at indicated time points. Viral mRNA (**a**) as well as cRNA and vRNA (**b**) of the influenza virus genome segment 5 were measured by a strand-specific RT-qPCR. The data shown represent the mean of three independent experiments and *error bars* indicate the standard deviation

In order to study the intracellular dynamics of influenza virus replication, we performed a strand-specific RT-qPCR on infected cells. This method enables the quantification of all three influenza virus RNA species, i.e., viral mRNA, cRNA, and vRNA. We measured the three RNA species of the genome segment 5, which encodes for the nucleoprotein. For this, additional cell culture flasks with MDCK cells were prepared and infected with the influenza virus PR8 at an MOI of 10 PFU per cell. The inoculum was removed after 1 h and fresh medium was added, but thereafter the medium was not replaced until cells were harvested. Viral mRNA levels increased directly after the infection and reached their maximal level around 4 hpi. Thereafter, viral mRNA levels steadily decreased (Fig. 3a). The increase of cRNA also occurred directly after the infection. From 4 hpi onwards, cRNA synthesis leveled out and the cRNA remained at a relatively low concentration (200 to 500 copies per cell) until the end of our infection experiment (Fig. 3b). For the vRNA, a higher starting concentration at 0 hpi was detected which is reasonable as infecting virus particles contain vRNA. In contrast, low levels of viral mRNA were detected at 0 hpi which most probably represents a contamination of the seed virus with viral mRNAs due to cell lysis during seed virus production. Similar to the synthesis of cRNA, vRNA levels reached a plateau from 4 hpi onwards, but vRNA concentrations were approximately two orders of magnitude higher than cRNA concentrations. In summary, viral RNA synthesis is initiated directly after the infection, but levels out already at 4 hpi.

### Imaging cytometry of virus-infected cells

**Fig. 4:**
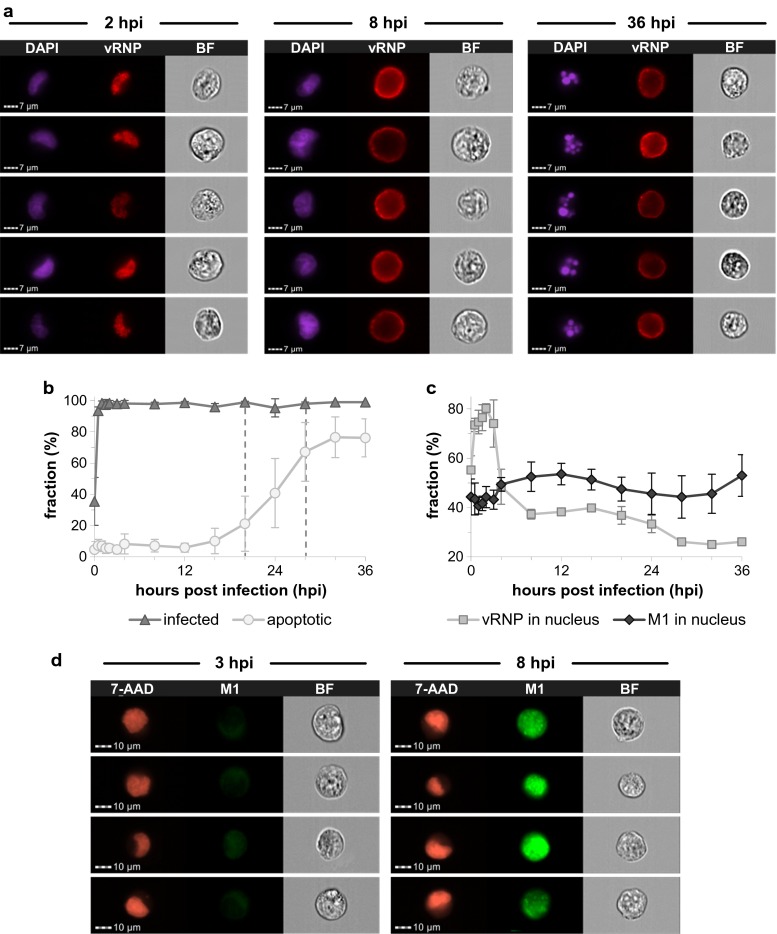
Imaging cytometry of virus-infected cells. MDCK cells were infected with influenza PR8 virus at an MOI of 10 PFU per cell. At indicated time points, cells were fixed and stained for vRNPs and cell nuclei using DAPI (**a**) or for M1 and cell nuclei using 7-AAD (**d**). **a** Images of individual cells at selected time points after the infection. Each panel series shows the DAPI, vRNP, and bright field (BF) signal of representative cells. **b, c** Spatio-temporal analysis of virus replication. Image analysis was performed using the IDEAS software. Diagrams show the following fractions (%): **b** Infected cells (showing a vRNP signal) and apoptotic cells (showing nuclear fragmentation); **c** vRNPs in the nucleus (based on the amount of vRNP signal co-localized with the DAPI signal) and M1 in the nucleus (based on the proportion of M1 signal co-localized with the 7-AAD signal). **d** Images of individual cells at selected time points after the infection. Each panel series shows the 7-AAD, M1, and BF signal of representative cells. *First and second dashed lines* indicate time points when ≥95 % of the final titer of infectious and total virus particles were released, respectively. *Error bars* indicate standard deviations of three independent experiments

Furthermore, we examined the progression of the infection by imaging cytometry, a method that combines the throughput of flow cytometry with the spatial resolution of fluorescent microscopy. Cells were stained using either a combination of DAPI and an antibody against vRNPs (Fig. 4a) or 7-AAD together with an antibody against M1 (Fig. 4d). Already 30 min after the high MOI infection, more than 90 % of the cell population contained vRNPs and within 1 h, almost the complete cell population was infected (Fig. 4b). At the later stage of the infection, condensation and fragmentation of the nucleus (DAPI) was observed (Fig. 4a, right panel). This staining pattern was used to identify apoptotic cells. The fraction of apoptotic cells started to increase between 16 and 20 hpi and reached its maximum of approximately 77 % at 32 hpi (Fig. 4b). In addition, we investigated the intracellular localization of vRNPs and M1. Between 30 min to 3 h after the infection, 70 to 80 % of the fluorescent signal of vRNPs was found in the nucleus (Fig. 4c). However, between 3 and 4 hpi, the proportion of the vRNP fluorescent signal intensity within the nucleus decreased strongly which indicates a fast nuclear export of vRNPs. Subsequently, vRNPs were mainly localized close to the plasma membrane as indicated by a ring-like staining pattern of vRNPs between 8 and 36 hpi (Fig. 4a). In contrast to vRNPs, M1 was more evenly distributed and not predominantly localized in the nucleus at early time points. Yet, between 3 and 4 hpi, the amount of M1 in the nucleus slightly increased (Fig. 4c). Due to the fact that the total signal intensity of M1 increased strongly in the course of the infection, this slight enrichment of M1 in the nucleus cannot be inferred easily from individual images (Fig. 4d). However, the analysis of thousands of cells clearly indicates that M1 is imported into the nucleus between 3 and 4 hpi while vRNPs are exported. Both shifts point to active transport processes. At later stages of the infection, however, the analysis of the nuclear localization of viral proteins was biased by the condensation and fragmentation of the nucleus caused by the induction of apoptosis. Therefore, the nuclear localization can be studied reliably only up to about 16 hpi. Taken together, imaging cytometry confirmed the rapid synchronized infection of the complete cell population and showed that a strong nuclear export of viral genomes occurred already between 3 and 4 hpi.

### Impact of cell cycle stage of MDCK cells on the influenza virus infection

**Fig. 5:**
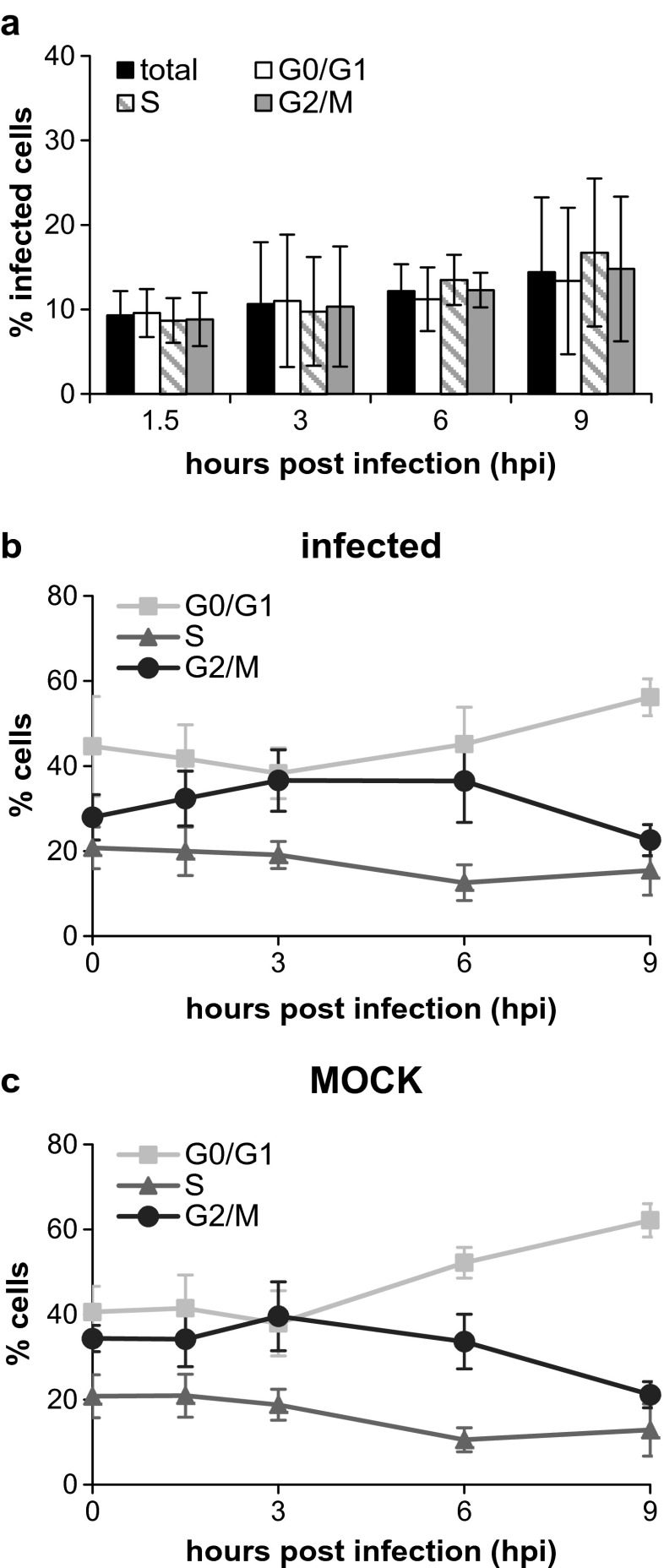
Evaluation of cell cycle and infection status. MDCK cells were infected with the influenza PR8 virus at an MOI of 0.1. Infection and cell cycle analysis were performed using imaging flow cytometry by staining for vRNPs and DAPI, respectively. **a** Comparison of the fraction of infected cells in the total population and in each cell cycle phase. **b** Proportion of infected cells in the different cell cycle phases. **c** Percentage of non-infected cells in the different cell cycle phases in mock cultures

As it was reported that influenza virus infects preferentially cells in the G_1_ phase of the cell cycle (Ueda et al. 2013) and that G_0_/G_1_ phase-synchronized cells produce higher virus titers compared to unsynchronized cells (He et al. 2010), we studied the impact of the cell cycle on influenza virus infections of MDCK cells. To this end, we infected MDCK cells with the influenza virus PR8 at an MOI of 0.1. At this low MOI, about 10 % of the cell population should become infected and we wanted to check whether these infected cells were predominantly in the G_1_ phase. The infection was performed for 45 min in serum-free medium containing trypsin. Subsequently, the inoculum was removed, cells were washed, and incubated in medium without trypsin but with 10 % FCS to avoid secondary infections and to reduce the impact of serum depletion on cell cycle progression. Based on the intensity of the DAPI staining, the cell cycle stage of cells was determined. In addition, an immunostaining for vRNPs was used to identify infected cells. As expected, about 10 % of the cell population were infected and this proportion stayed nearly constant for 9 h, indicating that almost no secondary infections occurred (Fig. 5a). Regardless of the cell cycle stage, in each subpopulation of cells, we found around 10 % infected cells, showing that cells in different cell cycle stages had a similar probability to become infected. Hence, we found no evidence that MDCK cells in a certain cell cycle stage are preferentially infected. Furthermore, infected MDCK cells showed a similar cell cycle progression compared to mock-infected cells within the first 9 hpi (Fig. 5b, c). This indicates that the infection did not induce a cell cycle arrest in the early phase of the infection. In summary, for MDCK cells, no impact of the cell cycle stage on the influenza virus entry was found.

## Discussion

For the optimization of cell culture-based influenza vaccine production, a thorough understanding of the influenza virus replication cycle and of virus-host cell interactions is crucial to develop strategies for a rational design of high yield production cell lines or vaccine strains. In this study, we analyzed in detail the influenza virus replication in MDCK cells. To this end, a high MOI infection was performed to examine a single round of infection in a synchronously infected cell population. An immunostaining against vRNPs and the analysis by imaging cytometry confirmed that the complete cell population was rapidly infected under these conditions (Fig. 4b). The progeny virus release was investigated by exchanging the complete supernatant of infected cultures with fresh medium every 4 h. In doing so, we were able to demonstrate that the virus release rate reached its maximum already in the sampling interval between 4 and 8 hpi (Fig. 1c). Moreover, we did not find a burst-like influenza virus production but instead a constant release of progeny virus particles over a relatively long period of time. To our knowledge, such a constant influenza virus release has not been reported before. In an early study, more fluctuations of the virus release have been found but sampling intervals were not as regular as in our experiment (Gaush and Smith 1968). Furthermore, we found that the ratio of infectious to total virus particles was stable during the early phase of the infection and below a ratio of 1:10 which is typical for influenza viruses (reviewed by Brooke (2014)). However, the release of infectious virions decreased approximately 8 h earlier than the production of particles measured in an HA assay (Fig. 1). The multitude of these noninfectious particles in the late phase of the infection (from 20 hpi onwards) probably did not result from the degradation of infectious particles since the experimental setup prevents extensive degradation by the repetitive harvest and the exchange of the complete culture supernatant every 4 h. Hence, in the late phase of the infection, cells seem to produce almost exclusively defective particles or cellular debris decorated with viral HA proteins, both able to contribute to the HA titer. Indeed, we found that the proportion of apparently intact spherical particles decreased in the late phase and more deformed or broken particles as well as large membranous structures were detected by electron microscopy (Fig. 2). It has been reported before that the morphology of noninfectious influenza virus particles differs from the uniform spherical shape of infectious particles by being pleomorphic, poorly defined, and in a state of disintegration (Paucker et al. 1959; Werner and Schlesinger 1954). Yet, these studies used noninfectious particles from undiluted passaging experiments in which the amount of infectious particles decreases due to the accumulation of so-called defective interfering particles (DIPs). In contrast, we observed that the particle morphology already changes in the course of one single infection cycle. In the late phase of the infection, cells predominantly produce pleomorphic noninfectious particles and large membranous structures. While DIPs were detectable throughout our infection experiment at high MOI, they did not accumulate predominantly in the late phase of the infection (data not shown). Therefore, it does not seem likely that DIPs cause the observed changes of the particle morphology and infectivity in the late phase. Yet, we found that these changes occurred in the phase when cell viability dropped, adherent cells started to detach from the bottom of the cell culture flask (Fig. 1d) and when nuclear fragmentation indicated apoptosis of infected cells (Fig. 4b). More research is required to understand why and when cells lose their capacity to produce infectious spherical particles and if this is associated with the progression of apoptosis. However, our results are in line with a previous report from our group showing that infectious particles are produced predominantly in the early phase during influenza vaccine production in bioreactors and that total virus release is terminated by cell death (Schulze-Horsel et al. 2009).

Changes of the particle morphology and infectivity in the late phase of the infection might have important consequences for vaccine manufacturing. Pleomorphic particles could be related to losses during purification steps such as size exclusion or affinity chromatography due to their variable shape and size. In addition, it has been reported by others that pleomorphic noninfectious particles contain reduced amounts of HA and vRNPs compared to infectious particles that are uniformly spherical (Paucker et al. 1959). Accordingly, noninfectious particles might yield lower antigen concentrations for split vaccines if they possess a reduced HA content. In addition, the accumulation of large membranous structures decorated with viral surface proteins might be a challenge for downstream purification regarding contaminant levels as it has been reported that such structures contain cellular enzymes and ribosomal RNA (Rott and Schafer 1961). Thus, these membranous structures can cause difficulties to achieve the depletion of cellular protein and nucleic acid contaminants which is crucial for the downstream processing of cell culture-based influenza vaccines. Furthermore, noninfectious particles have been described to possess certain biological activities (Brooke 2014; Marcus et al. 2009) and their production in the late phase of infection can affect the efficacy and quality of live-attenuated vaccines even more dramatically. Hence, optimization of the harvesting time point should be performed carefully taking the characteristics of noninfectious particles into account.

Our analysis of the intracellular dynamics of influenza virus replication shows that the synthesis of all three viral RNA species occurs early during the infection and levels out already at 4 hpi (Fig 3). This raises the question why viral RNA synthesis shuts down so early and whether a prolonged phase of viral RNA synthesis would lead to higher cell-specific virus yields. Two influenza virus proteins have been reported to inhibit viral RNA synthesis, i.e., M1 and NEP. It was recognized early that the M1 protein inhibits influenza virus polymerase activity (Zvonarjev and Ghendon 1980) similar to the matrix proteins of other negative-strand RNA viruses (reviewed by Kranzusch and Whelan (2012)). However, it was shown recently that NEP constitutes an additional regulator of influenza virus RNA synthesis (reviewed by Paterson and Fodor (2012)). In particular, NEP recruits M1 to vRNPs, contributes to the shutdown of RNA synthesis, and enables the nuclear export of vRNPs (Brunotte et al. 2014). We could show by imaging cytometry that M1 accumulates in the nucleus (Fig. 4c) at the same time when viral RNA synthesis decreases (Fig. 3). However, it is not clear yet if viral RNA synthesis decreases due to the binding of M1 and/or NEP to vRNPs or due to M1 and/or NEP-mediated nuclear export of viral genomes, which removes the template for mRNA and cRNA synthesis. In fact, we also observed a strong export of vRNPs from the nucleus into the cytoplasm between 3 and 4 hpi (Fig. 4c) which coincides with the decrease in viral RNA synthesis (Fig. 3). Thus, both the accumulation of regulatory viral proteins and the nuclear export of viral genomes might contribute to the early shutdown of viral RNA synthesis. In this regard, influenza virus replication might be a self-limiting process. In particular, the accumulation of regulatory viral proteins terminates genome replication rather early and induces the transport of viral genomes towards assembly and budding sites within the host cell. However, our results indicate that additional bottlenecks at later stages of the influenza life cycle exist which might limit the release of progeny virus. On the one hand, we found a relatively long period in which cells release virus particles with a constant rate (Fig. 1c). On the other hand, we observed an accumulation of vRNPs in the proximity of the plasma membrane (Fig. 4a). Thus, the nuclear export of viral genomes occurs already early during the infection cycle but the release of virions, which requires transport of viral proteins and genomes to the plasma membrane, virus assembly, and budding, seems to comprise unknown rate limiting steps.

It has been reported by others that the influenza virus preferentially infects human H292 lung epithelial cells in the G1 phase (Ueda et al. 2013). In contrast, our infection of a MDCK cell population at low MOI showed no preferential infection in any cell cycle stage (Fig. 5). Ueda et al. used the same influenza virus strain (PR8) but cell lines differed which might explain contradictory results. In addition, the experimental setup was different since they used a single-virus infection system. For human A549 cells, it has been shown that influenza virus infections cause a cell cycle arrest in the G_0_/G_1_ phase and cells synchronized in the G_0_/G_1_ phase showed increased viral protein expression as well as higher progeny virus production (He et al. 2010). In MDCK cells, we did not find higher viral protein expression of cells in the G_0_/G_1_ phase based on the fluorescent intensity of the imaging flow cytometry measurement (data not shown). Furthermore, no cell cycle arrest in the early phase of the infection up to 9 h was observed (Fig. 5b, c). Unfortunately, we were not able to analyze later time points since we found secondary infections at 12 hpi even in the absence of trypsin. However, the result that the cell cycle stage has no impact on influenza virus replication in MDCK cells is in line with our previous observation that the cell-specific virus yield does not correlate to the cell size while cell size itself is typically correlated to the cell cycle stage (Heldt et al. 2015).

In conclusion, the systematic analysis of the influenza virus propagation in cell culture identified two stages of the virus replication that might limit cell-specific virus yields in vaccine manufacturing. On the one hand, our results suggest that influenza virus replication is a self-limiting process. In particular, the shutdown of viral genome replication and the transport of viral genomes towards assembly and budding sites is induced very early during the infection cycle. However, a prolonged viral RNA synthesis might lead to higher virus yields. This replication strategy might be disadvantageous in nature as influenza viruses have to outrun the host immune response, but could be superior in the artificial setting of cell culture-based virus production. On the other hand, additional bottlenecks seem to exist in the late phase of the viral life cycle since progeny virus release occurs at a rather constant rate for a relatively long period until cells become apoptotic. Eventually, the identification of rate-limiting steps during influenza virus replication might not only support the optimization of vaccine manufacturing but also provides promising targets for the development of new antivirals.

## Electronic supplementary material

ESM 1 (PDF 147 kb)
