# Supplementary material for: Influenza virus intracellular replication dynamics, release kinetics, and particle morphology during propagation in MDCK cells
Source: Appl Microbiol Biotechnol. 2016 Apr 29;100:7181–92. doi: 10.1007/s00253-016-7542-4 (PMC4947482; doi:10.1007/s00253-016-7542-4)
Supplement: Supplementary file 1 — (PDF 147 kb) [file 253_2016_7542_MOESM1_ESM.pdf]

Applied Microbiology and Biotechnology

Influenza virus intracellular replication dynamics, release kinetics and particle morphology during propagation in MDCK cells

Timo Frensing[1,2*‡], Sascha Kupke[1‡], Mandy Bachmann[1‡], Susanne Fritzsche[1], Lili Gallo-Ramirez[1], Udo Reichl[1,2]

[1] Bioprocess Engineering, Max Planck Institute for Dynamics of Complex Technical Systems, Sandtorstrasse 1, 39106 Magdeburg, Germany

[2] Chair of Bioprocess Engineering, Otto-von-Guericke University Magdeburg, Universitätsplatz 2, 39106 Magdeburg, Germany

[‡] contributed equally

* corresponding author:

Jun.-Prof. Dr. Timo Frensing
Max Planck Institute for Dynamics of Complex Technical Systems
Bioprocess Engineering
Sandtorstrasse 1; 39106 Magdeburg; Germany
E-mail: frensing@mpi-magdeburg.mpg.de
Phone: +49 391 6110 332
Fax: +49 391 6110 598

Tab. S1: Primer sets for RNA reference standard 5 generation.

RNA-type	Primer name	Sequence (5´-3´)	Position (nt)
cRNA	Seg 5 Uni T7 for	TAATACGACTCACTATAGGGAGCAAAAGCAGGGTAGATAATC	1 - 22
	Seg 5 Uni rev	AGTAGAAACAAGGGTATTTTTC	1543 - 1565
vRNA	Seg 5 Uni for	AGCAAAAGCAGGGTAGATAATC	1 - 22
	Seg 5 Uni T7 rev	TAATACGACTCACTATAGGGAGTAGAAACAAGGGTATTTTTC	1543 - 1565
mRNA	Seg 5 Uni T7 for	TAATACGACTCACTATAGGGAGCAAAAGCAGGGTAGATAATC	1 - 22
	Seg 5 dT rev	TTTTTTTTTTTTTTTTCTTTAATTGTC	1533 - 1549

Tab. S2: Primers used for the reverse transcription of specific influenza virus RNA species.

Target	RNA-	Primer name	Sequence (5´-3´)	Position (nt)
Segment 5	cRNA	Seg 5 tagRT rev	GCTAGCTTCAGCTAGGCATCAGTAGAAACA AGGGTATTTTTCTT	1541 - 1565
	vRNA	Seg 5 tagRT for	ATTTAGGTGACACTATAGAAGCGAGTGATT ATGAGGGACGGTTGAT	192 - 215
	mRNA	Oligo tagdTRT	GTAAAACGACGGCCAGTTTTTTTTTTTTTTT	-

Tab. S3: Primers used for real-time RT-qPCR.

Target		Primer name	Sequence (5´-3´)	Position (nt)
Introduced tag sequence	vRNA	vRNA tagRealtime for	ATTTAGGTGACACTATAGAAGCG	-
	cRNA	cRNA tagRealtime rev	GCTAGCTTCAGCTAGGCATC	-
	mRNA	mRNA tagRealtime rev	GTAAAACGACGGCCAGT	-
Segment 5	vRNA	Seg 5 Realtime rev	CGCACTGGGATGTTCTTC	282 - 300
	c/mRNA	Seg 5 Realtime for	GGAAAGTGCAAGACCAGAAGAT	1388 - 1410

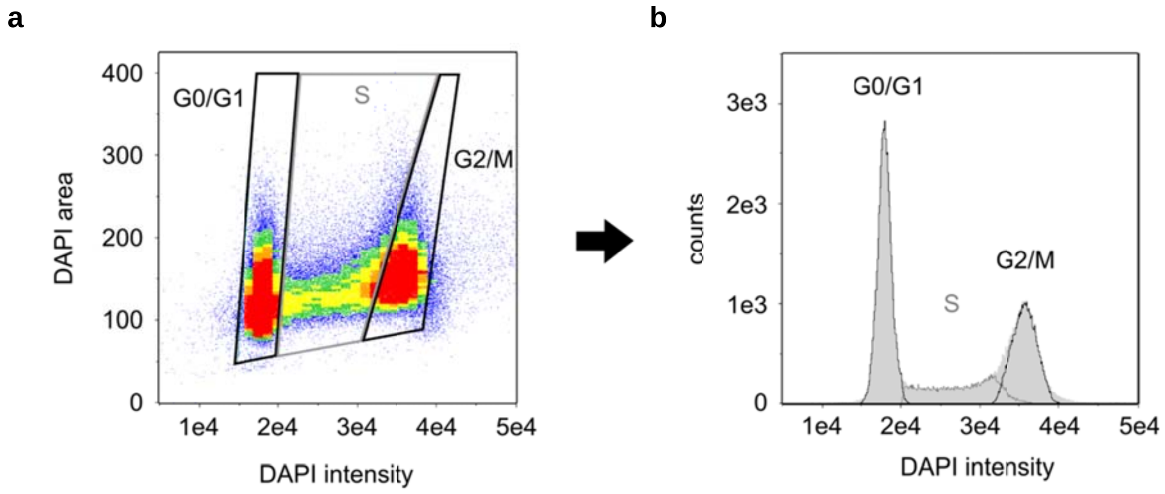

Fig. S1 Gating of cell cycle phase populations of DAPI-stained MDCK cells using the ImageStream X system. (**a**) The DAPI intensity (sum of grey values of each pixel within the object) of single, focussed cells was plotted against the corresponding area of the DAPI signal. The cell cycle gates for the G0/G1 phase and G2/M phase were adjusted according to the expected DNA content. For this, the two main populations (mean DAPI intensity differs by the factor of two) were selected in the intensity vs. area dot plot taking also into acount their slightly tilted distribution. The cells in between these two well-defined populations were assigned to the S phase. (**b**) Histograms of the gated cell cycle populations plotted (solid lines) on top of the total single cell population (light grey filled area).
